# Echocardiographic and Biochemical Factors Predicting Arrhythmia Recurrence After Catheter Ablation of Atrial Fibrillation—An Observational Study

**DOI:** 10.3389/fphys.2019.01215

**Published:** 2019-10-02

**Authors:** Emmanouil Charitakis, Lars O. Karlsson, Joanna-Maria Papageorgiou, Ulla Walfridsson, Carl-Johan Carlhäll

**Affiliations:** ^1^Department of Cardiology and Department of Medical and Health Sciences, Linköping University, Linköping, Sweden; ^2^Division of Cardiovascular Medicine and CMIV, Linköping University, Linköping, Sweden; ^3^Department of Clinical Physiology and Department of Medical and Health Sciences, Linköping University, Linköping, Sweden

**Keywords:** atrial fibrillation, radiofrequency ablation, natriuretic peptides, left atrial emptying fraction, fibrosis, apoptosis

## Abstract

**Background:** RFA is a well-established treatment for symptomatic patients with AF. However, the success rate of a single procedure is low. We aimed to investigate the association between the risk of recurrence of atrial fibrillation (AF) after a single radiofrequency ablation (RFA) procedure and cardiac neurohormonal function, left atrial (LA) mechanical function as well as proteins related to inflammation, fibrosis, and apoptosis.

**Methods and Results:** We studied 189 patients undergoing RFA between January 2012 and April 2014, with a follow-up period of 12 months. A logistic regression analysis was performed to investigate the association between pre-ablation LA emptying fraction (LAEF), MR-proANP, Caspase-8 (CASP8), Neurotrophin-3 (NT3), and the risk for recurrence of AF after a single RFA procedure. 119 (63.0%) patients had a recurrence during a mean follow-up of 402 ± 73 days. An increased risk of recurrence was associated with: Elevated MR-proANP (fourth quartile vs. first quartile: HR, 2.80 (95% CI, 1.14–6.90]; *P* = 0.025); Low LAEF (fourth quartile vs. first quartile: hazard ratio [HR], 2.41 [95% CI, 1.01–5.79]; *P* = 0.045); Elevated CASP8 (fourth quartile vs. first quartile: HR 12.198 95% CI 2.216–67.129; *P* = 0.004); Elevated NT-3 (fourth quartile vs. first quartile: HR 7.485 95% CI 1.353–41.402; *P* = 0.021). In a receiver operating characteristic curve analysis, the combination of MR-proANP, CASP8, and NT3 produced an area under the curve of 0.819; CI 95% (0.710–0.928).

**Conclusions:** Patients with better LA mechanical function and lower levels of atrial neurohormones as well as of proteins related to fibrosis and apoptosis, have a better outcome after an RFA procedure.

**Unique identifier:** No. NCT01553045 (https://clinicaltrials.gov/ct2/show/NCT01553045?term=NCT01553045&rank=1).

## Introduction

Radiofrequency ablation (RFA) has become a mainstream treatment option for symptomatic patients with drug refractory atrial fibrillation (AF) (Haissaguerre et al., 1998; Kirchhof et al., 2016). Freedom from arrhythmia after RFA can depend on factors related to the patients, as well as technical aspects of the procedure. Success rates after a single RFA procedure varies widely, ranging from 30 to 80% (Cheema et al., 2006; Sultan et al., 2017). An explanation for this extreme variation can be the heterogenicity of the patient population that suffer from AF, with respect to gender, age, type of AF and presence of structural heart disease. Furthermore, cardiac endocrine imbalance and comorbidities such as obesity, hypertension, diabetes mellitus, or active inflammatory disease may contribute to the disparity.

Given the moderate success rate of RFA, identifying predictors of success in maintaining sinus rhythm (SR) after a single RFA procedure can facilitate patients' selection for RFA with a favorable outcome, and at the same time reduce the risk of exposing patients to unnecessary procedures and related complications (Balk et al., 2010).

The SMURF-study (Symptom burden, metabolic profile, cardiac imaging, rhythm, neurohormonal activation, hemodynamics, and health-related quality of life in patients with AF) was an observational, single-center cohort study conducted between January 2012 and April 2014 (Charitakis et al., 2015).

SMURF-study showed that anxiety, depression, low-grade inflammation were the factors that predicted both arrhythmia-related symptoms and HRQoL. Obesity was the most significant predictor of patients' general health (Charitakis et al., 2017). With regards to the effect of RFA on cardiac biomarkers, the SMURF-study showed that RFA of AF is a strong stimulus with direct and significant impact on different neurohormonal systems (Charitakis et al., 2016). In the context of the SMURF-study, it was also found that factors such as LA volume, gender, the frequency of attacks prior to RFA and recurrences of AF after RFA significantly affected the improvement in symptoms and HRQoL in patients with AF after RFA (Barmano et al., 2019a). Finally, in male patients with AF, alcohol consumption was associated with higher levels of natriuretic peptides, and the need for re-ablation (Barmano et al., 2019b).

The objective of the present study was to investigate the association between the risk of recurrence after a single RFA for AF and metrics of left atrial (LA) mechanical function, markers of cardiac endocrine function, as well as proteins related to inflammation, fibrosis and apoptosis. The maximum LA volume (LAV_max_) and the LA emptying fraction (LAEF) was measured in order to assess the LA size and mechanical function. In order to assess the endocrine function of the heart, plasma concentrations of the mid-regional fragment of the N-terminal of pro-atrial natriuretic peptide (MR-proANP) and the N-terminal of pro B-natriuretic peptide (NT-proBNP) were measured. The level of inflammation, fibrosis and apoptosis was assessed by the concentrations of caspase 8 protein (CASP8), neurotrophin-3 (NT3) protein, and high-sensitive C-reactive protein (hsCRP). This objective has not been evaluated previously in the context of the SMURF-cohort.

## Methods

### Population

Patients referred for RFA due to AF at the University Hospital in Linköping, Sweden, were considered for participation *in the SMURF-study*. The inclusion criteria were: (1) Age ≥18 years with paroxysmal or persistent AF, (2) Patients referred for first time RFA, and (3) Patients with sufficient knowledge of the Swedish language.

Exclusion criteria were (1) Patients with previous or expected heart surgery, (2) Patients with severe heart failure with left ventricular ejection fraction (LVEF) <35%, or 3) Patients with acute coronary syndrome during the past 3 months. The full study protocol has been published previously (Charitakis et al., 2015).

### Informed Consent and Ethical Considerations

The Regional Ethical Review Board of the Faculty of Health Sciences, Linköping, Sweden, approved the protocol for this study (2011/40–31, 2012/226–32). All patients gave their written consent and the study complies with the Declaration of Helsinki (Rickham, 1964).

### Subject Measurements

The subject measurements have been described previously (Charitakis et al., 2015). In brief, all patients were subjected to a full baseline evaluation including medical history, physical examination, 12-lead ECG, heart CT scan and transthoracic echocardiographic examination (TTE) the day before the procedure.

Patients were catheterized, and blood samples were drawn from a peripheral vein, and the coronary sinus (CS) *directly after catheterization and before the ablation procedure*. The ablation procedure has been described previously (Charitakis et al., 2015).

### Echocardiography

All participants underwent TTE prior to the RFA procedure. GE Vivid 7 or GE Vivid E9 system (GE Healthcare, Horten, Norway) were utilized with a 3.5-MHz transducer

The LVEF was calculated according to Simpson's biplane method. The LA volume was measured using the biplane area-length method. The LAV_max_ was measured just before mitral valve (MV) opening and corrected for body surface area (BSA). The LA minimum volume (V_min_) was measured immediately after MV closure (Vizzardi et al., 2012). LAEF was calculated using the following equation (V_max_-V_min_)/ V_max_ × 100 (Vizzardi et al., 2012; Chou et al., 2018).

### Laboratory Tests

The concentration of NT-proBNP was analyzed on the Elecsys 2010 platform (Roche Diagnostics, Mannheim, Germany). The total coefficient of variation (CV), was 4.6% at 426.5 pg/ml (*n* = 487) and 3.2% at 2,308 pg/ml (*n* = 495). Plasma concentration of MR-proANP was analyzed on the Kryptor platform (Brahms AG, Hennigsdorf Germany). The intraassay CV for MR-proANP according to the manufacturer was ≤5% for concentrations between 10 pmol/l and 20 pmol/l, <3.5% for concentrations between 20 and 1,000 pmol/l, and <3.5% for concentrations over 1,000 pmol/l.

The high-sensitivity C-reactive protein (hsCRP) concentration analysis was performed using the wide range-C-Reactive Protein immunoturbidimetric assay on the ADVIA 1,650 system (Siemens Healthcare Gmbh, Erlangen, Germany) as previously described (Charitakis et al., 2017).

Blood samples for the analysis of CASP8 and NT3 were collected from the CS and peripheral blood. CAPS8 and NT3 were analyzed, using the proximity extension assay (PEA) with the Proseek Multiplex Inflammation panel (Olink Proteomics AB, Uppsala, Sweden) at the Clinical Biomarkers Facility, Science for Life Laboratory, Uppsala (Assarsson et al., 2014). This panel includes 92 biomarkers. CASP8 and NT3 were expressed in normalized protein expression (NPX) values, which is an arbitrary unit on a log2 scale in which a high value corresponds to high protein expression.

### Follow-Up and Definition of Recurrence

Patients' follow-up was conducted 4 and 12 months after RFA and whenever required due to symptoms of AF. 12-lead electrocardiograms and 24 h Holter ambulatory electrocardiograms were recorded at 4 and 12 months and when patients exhibited AF-related symptoms. Episodes of AF or atrial tachyarrhythmia lasting more than 30 s during the follow up period on 12-lead electrocardiograms, 24 h Holter ambulatory or on pacemaker/implantable defibrillator interrogation were classified as clinical recurrences (Calkins et al., 2012).

### Endpoint

The primary endpoint of the study was the possible association between parameters of biochemical analyses (concentrations of NT-proBNP, MR-proANP, CASP8, NT3, and hsCRP), echocardiography (LAV_max_, LAEF), and the risk for recurrence after RFA of AF.

### Statistical Methods

For baseline data, continuous variables were expressed as means ± standard deviation (SD) and variables not normally distributed as medians with 25th and 75th percentiles within brackets. Categorical data were presented as counts with percentages within brackets. Patients with missing values were excluded from the planned analyses.

Analyses were calculated using an unpaired *t*-test for continuous variables; the chi -test was used for categorical variables. Evaluation of correlation was analyzed using the Pearson's correlation coefficient.

Patients were stratified into quartiles of MR-proANP, NT-proBNP, hsCRP, CASP8 NT3 concentrations, LAV_max_ and LAEF, providing better sensitivity than the alternative approach sometimes used, i.e., classifying values as above or below the median.

Multiple logarithmic regression analyses were performed to determine the predictors of arrhythmia recurrence after RFA. The presence of any arrhythmia recurrence during the first 12 months after the first RFA of AF was used as a dependent variable. MR-proANP, NT-proBNP, hsCRP, CASP8, NT3 concentrations, LAV_max_, and low LAEF were used as independent predictors of arrhythmia recurrence after RFA of AF. The analyses were adjusted for covariates: age>65 years, gender, BMI >30 kg/m^2^, type of AF (paroxysmal or persistent), rhythm at the time of blood sample retrieval (SR or AF), LVEF <50%, glomerular filtration rate of <60 ml/min/1.73 m^2^ (Grubb et al., 2005) and the use of renin-angiotensin-aldosterone system (RAAS) inhibitors furosemide and b-blockers.

The models were fit by an enter method, where all variables were entered the original model. Variables with *p*-values >0.1 were thereafter removed.

To evaluate the possible prognostic value of the different tested predictors in a multivariate analysis, a weighted variable was produced based on the beta-value in the multivariate logarithmic regression analysis. A receiver operating characteristic (ROC) curve analysis was then performed (DeLong et al., 1988).

In order to validate the multivariable logistic regression models, analysis of residuals and multicollinearity diagnostics were performed.

All reported *p*-values were two-sided and a *p*-value <0.05 was considered statistically significant. The analyses were performed using the SPSS 24.0 (SPSS, Chicago, IL, USA).

## Results

**Figure 1:**
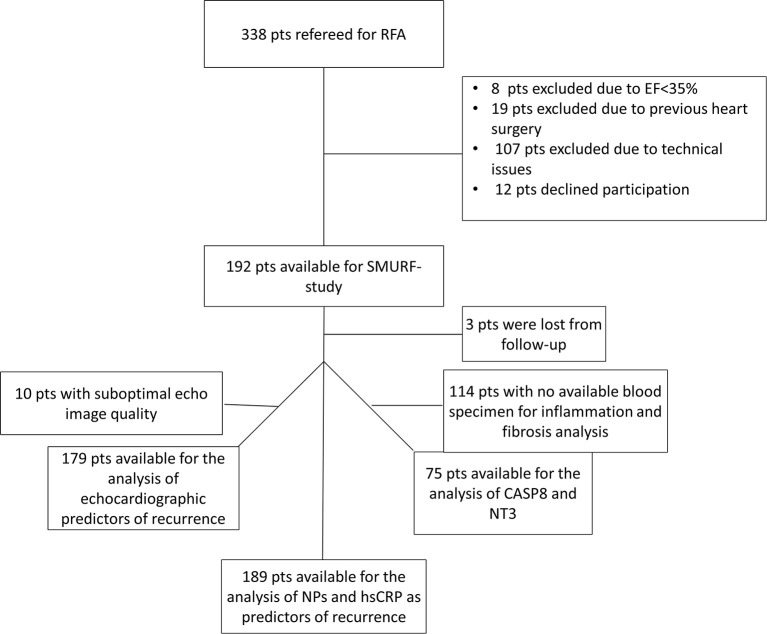
Flow chart of the study participation inclusion. AF, atrial fibrillation; CASP8, caspase 8EF: ejection fraction; pats, patients; hsCRP, high sensitive C-reactive protein; RFA, radiofrequency ablation; NP, natriuretic peptides; NT3, neurotrophin-3.

A total of 192 participants undergoing RFA were included in the SMURF study (56 women and 136 men), 3 patients were lost from follow-up, and a total of 189 participants were evaluated for the primary endpoint (Figure 1). The baseline characteristics are presented in detail in Table 1.

**Table 1:** Characteristics of all patients at baseline, and of patients with or without AF recurrence during the 1 year follow up period.

	**All patients**	**AF recurrence**	**No AF recurrence**	***p*-value**
Number of pts	189	119 (63%)	70 (37%)	
Female	55 (29.1%)	35 (29.4%)	20 (28.6%)	0.902
Male	134 (70.9%)	84 (70.6%)	50 (71.4%)	0.902
Age	60.5 ± 10.3	61 ± 9.3	59.6 ± 11.8	0.388
Hypertension	80 (42.3%)	50 (42%)	30 (43%)	0.91
Diabetes	16 (8.5%)	11 (9.2%)	5 (7.1%)	0.879
BMI	27.4 (22.6, 34.2)	27.5 (21.6, 33.4)	26.8 (21.5, 32.1)	0.254
CKD (GFR <60 mL/min/1.73 m^2^)	40 (21.2%)	24(20.1%)	16 (22.9%)	0.662
Stroke/TIA	11 (5.8%)	8 (6.7%)	3 (4.3%)	0.49
IHD	15 (7.9%)	9 (7.5%)	6 (8.6%)	0.561
CHA_2_DS_2_VASc score	2 (0, 5)	2 (0, 5)	1.5 (0, 4.5)	0.902
Beta blocker	139 (73.5%)	92 (77.3%)	47(67.1%)	0.126
AAD	98 (51.9%)	63 (53%)	35 (50%)	0.696
statins	54 (28.6%)	36 (30.3%)	18 (25.7%)	0.505
RAAS inhibitors	110 (58.2%)	70 (58.8%)	40 (57.1%)	0.821
LVEF <50%	49 (25.9%)	33 (27.8%)	16 (22.9%)	0.46
Paroxysmal AF	71 (37.6%)	41 (34.5%)	30 (42.9%)	0.249
Persistent AF	119 (63%)	78 (65.5%)	41 (58.6%)	0.249
MR-proANP (pmol/L)	135.3 (92.3, 189.7)	138.3(13.6, 263)	118.3 (28.1, 208.4))	0.040
NT-proBNP (mg/mL)	170 (72, 480)	170 (0, 632)	165(0, 519.3)	0.0468
LAV_max_/BSA (ml/m^2^) (*n =* 179)	27 (22-32)	27 (17, 37)	26 (16.8, 35.3)	0.139
LAEF (%) (*n =* 179)	46.3 (31.7, 55.8)	43.5 (16.5, 67.5)	49.2 (30.2, 68.2)	0.027
hsCRP (mg/L)	1.05 (0.4, 2,7)	1.1 (0, 3.1)	1 (0, 3)	0.349
CASP8 (*n =* 75)	1.39 ± 0.46	1.49 ± 0.45	1.23 ± 0.44	0.015
NT3 (*n =* 66)	1.7 ± 0.25	1.74 ± 0.25	1.63 ± 0.24	0.064

*Normally distributed continuous data are presented as means with standard deviation and differences examined with t-test non-parametric data are presented as median values with 25th and 75th percentiles within brackets and tested with Mann-Whitney U-test, categorical data are presented as counts with percent values within brackets and tested with chi-square test*.

*AAD, antiarrhythmic drugs; AF, atrial fibrillation; BMI, body mass index; BSA, body mass index; CASP8, caspase 8; CHA_2_DS_2_ VASc, congestive heart failure, hypertension, age ≥ 75, diabetes, stroke, vascular disease, age ≥ 65, sex; CKD, chronic kidney failure; LVEF, left ventricular ejection fraction; GFR, glomelular filtration rate; IHD, ischemic heart disease; LA EF, left atrial emptying fraction; LAV_max_, maximum left atrial volume; MR-proANP, mid-regional fragment of the N-terminal precursor of atrial natriuretic peptide; NT-3, Neurotrophin 3; NT-proBNP, N-terminal pro B-type natriuretic peptide. RAAS: renin-angiotensin II-aldosterone system*.

At the time of baseline blood sampling, 51 out of 189 participants were in AF. The mean RFA procedural time was 188 ± 49 min and the total RFA time was 40 ± 13 min. Additional ablation lines were performed in 27 participants.

Complications occurred in seven participants (3.7%). Two participants suffered from cardiac tamponade requiring pericardiocentesis, and one participant suffered from pericardial effusion not requiring pericardial drainage. Furthermore, three participants developed pseudoaneurysm, and another patient developed a larger than normal hematoma of the groin.

A total of 119 (62%) patients suffered a recurrence after a single RFA procedure during a mean follow up period of 402 ± 73 days. Fifty-eight (30.7%) patients were subjected to re-ablation and 23 (12.1%) patients remained on antiarrhythmic drugs. Furthermore, 53% of patients reported no arrhythmia-related symptoms at 12 months follow-up.

### Neurohormonal Predictors of Recurrence

During a follow up period of 402 ± 73 days, 78% of patients with high MR-proANP concentration [4th quartile (Q)] and 56% of patients with low MR-proANP concentration (1st Q) had suffered a recurrence. This difference was statistically significant (*p* = 0.023). Patients with high MR-proANP concentration had 2.8 times higher risk for recurrence compared with those with low MR-proANP concentration (*p* = 0.025; Table 2). No difference in risk for recurrence was observed between patients with high and low concentrations of NT-proBNP (*p* = 0.403; Table 2).

**Table 2:** Neurohormonal predictors of recurrence after RFA of AF.

***N* = 189**	**Hazard ratio**	**95% CI**	***p*-value**
**MR-proANP baseline (pmol/L)**			
2 (92.3–135.3)	1.327	0.580–3.033	0.503
3 (135.4–189.7)	0.955	0.428–2.127	0.955
**4 (>189.7)**	**2.8**	**1.135–6.910**	**0.025**
**NT-proBNP baseline (pg/mL)**			
2 (72–170)	1.277	0.558–2.923	0.562
3 (170.1–480)	1.000	0.439–2.279	1.000
4 (>480)	1.402	0.600–2.279	0.435

*Results of multivariable logistic regression analyses*.

*Data are presented in quartiles with first quartile as a reference*.

*Analyses were performed with multivariable logistic regression method and were adjusted for covariates: age>65 years, gender, BMI >30 kg/m^2^, type of AF (paroxysmal or persistent), rhythm at the time for blood sample retrieval (SR or AF), left ventricular ejection fraction <50% and glomerular filtration rate of <60 ml/min/1.73 m^2^. The potential statistical significances of those analyses are presented with P-values. Statistical significance in bold font*.

*AF, atrial fibrillation; BMI, body mass index; CI, confidence interval; MR-proANP, mid-regional fragment of the N-terminal precursor of atrial natriuretic peptide; NT-proBNP, N-terminal pro B-type natriuretic peptide; RFA, radiofrequency ablation; SR, sinus rhythm*.

### Echocardiographic Predictors of Recurrence

Regarding echocardiographic predictors of recurrence, patients with high LAEF (4th Q) suffered a significant lower percentage of recurrences after RFA of AF (48.8%) compared with those with low LA EF (1st Q: 69.7%, *p* = 0.046; 2nd Q: 71.1% *p* = 0.031). Moreover, LAEF showed a significant prognostic power, as patients in the 1^st^ and 2nd Q of LAEF, had a 2.4 and 2.5 higher risk for recurrence, respectively, compared with patients in the 4th Q (2nd vs. 4th Q *p* = 0.033; 1st vs. 4th Q *p* = 0.048) (Table 3). The LAV_max_ corrected for BSA did not rise as a significant prognostic factor (*p* = 0.246; Table 3).

**Table 3:** Echocardiographic predictors of recurrence after RFA of AF.

***N* = 179**	**Hazard ratio**	**95% CI**	***p*-value**
**LAVmax/BSA (ml/m**^**2**^**)**			
3 (27.1–32)	0.996	0.412–2.406	0.992
2 (22.1–27)	0.714	0.296–1.722	0.714
1 (12–22)	0.583	0.246.1.385	0.246
**LA EF (%)**			
3 (46.4–55.8)	1.783	0.772–4.117	0.175
**2 (31.8–46.3)**	**2.573**	**1.078–6.144**	**0.033**
**1 (27-31.7)**	**2.413**	**1.006–5.786**	**0.048**

*Results of multivariable logistic regression analyses*.

*Data are presented in quartiles with fourth quartile as a reference*.

*Analyses were performed with multivariable logistic regression method and were adjusted for covariates: age >65 years, gender, BMI >30 kg/m^2^, type of AF (paroxysmal or persistent), rhythm at the time of blood sample retrieval (SR or AF), left ventricular ejection fraction <50, glomerular filtration rate of <60 ml/min/1.73 m^2^ and the use of Renin-angiotensin-aldosterone system (RAAS) inhibitors, furosemide and b-blockers. The potential statistical significances of those analyses are presented with P-values. Statistical significance in bold font*.

*AF, atrial fibrillation; BMI, body mass index; BSA, body mass index; CI, confidence interval; LA EF, left atrial emptying fraction; LAV_max_, maximum left atrial volume; RFA, radiofrequency ablation; SR, sinus rhythm*.

### Inflammatory/Fibrosis/Apoptosis Predictors of Recurrence

Regarding markers of inflammation and apoptosis, 84% of patients with high CASP8 concentration in CS blood (CASP8_CS_) (4th Q) had a recurrence, whereas only 39% of patients with low CASP8_CS_ concentration (1st Q) suffered a recurrence (*p* = 0.027). Patients with high CASP8_CS_ concentration had more than 12 times higher risk for recurrence compared with patients with low CASP8_CS_ concentration(*p* = 0.004; Table 4).

**Table 4:** Inflammatory, fibrosis and apoptosis predictors of recurrence after RFA of AF.

	**Hazard ratio**	**95% CI**	***p*-value**
**hsCRP (mg/L) (*****n*** **= 189)**			
2 (0.4–1.05)	1.036	0.436–2.462	0.937
3 (1.06–2.7)	1.495	0.616–3.626	0.374
4 (>2.7)	0.667	0.293–1.516	0.333
**CASP8 (*****n*** **= 75)**			
2 (1.13–1.33)	2.622	0.646–10.634	0.177
3 (1.34–1.65)	1.987	0.504–7.841	0.327
**4 (>1.65)**	**12.198**	**2.216-67.129**	**0.004**
**NT3 (*****n*** **= 66)**			
**2 (1.49–1.66)**	**8.255**	**1.48–46.028**	**0.016**
**3 (1.67–1.85)**	**13.677**	**2.242–83.432**	**0.005**
**4 (>1.86)**	**7.485**	**1.353–41.402**	**0.021**

*Results of multivariable logistic regression analyses*.

*Data are presented in quartiles with first quartile as a reference*.

*Analyses were performed with multivariable logistic regression method and were adjusted for covariates: age > 65 years, gender, BMI >30 kg/m^2^, type of AF (paroxysmal or persistent), rhythm at the time of blood sample retrieval (SR or AF), left ventricular ejection fraction <50%, glomerular filtration rate of <60 ml/min/1.73 m^2^ and the use of Renin-angiotensin-aldosterone system (RAAS) inhibitors, furosemide and b-blockers. The potential statistical significances of those analyses are presented with P-values. Statistical significance in bold font*.

*AF, atrial fibrillation; BMI, body mass index; CASP8, caspase 8; CI, confidence interval; NT3, neurotrophin-3; RFA, radiofrequency ablation; SR, sinus rhythm*.

In case of markers of inflammation and fibrosis, patients with low NT3 concentration in CS blood (NT3_CS_) (1st Q) suffered a significant lower percentage of recurrences (29.4%) compared with those with higher NT3_CS_ concentration (2nd Q: 68.75%, *p* = 0.028; 3rd Q: 81.25%, *p* = 0.005; 4th Q:64.7, *p* = 0.044). In addition, NT3_CS_ was as a significant predictor of recurrence since patients with high NT3_CS_ concentration had more than seven times higher risk for recurrence compared with patients with low NT3_CS_ concentrations(*p* = 0.0021; Table 4).

In contrast, hsCRP, CAPS8, and NT3 in peripheral blood were not significant predictors of recurrence after RFA of AF (hsCRP: *p* = 0.333; CASP8_pb_ *p* = 0.503; NT-3_pb_: *p* = 0.172; Table 4).

### Prognostic Value of a Multi-Predictor Model

**Figure 2:**
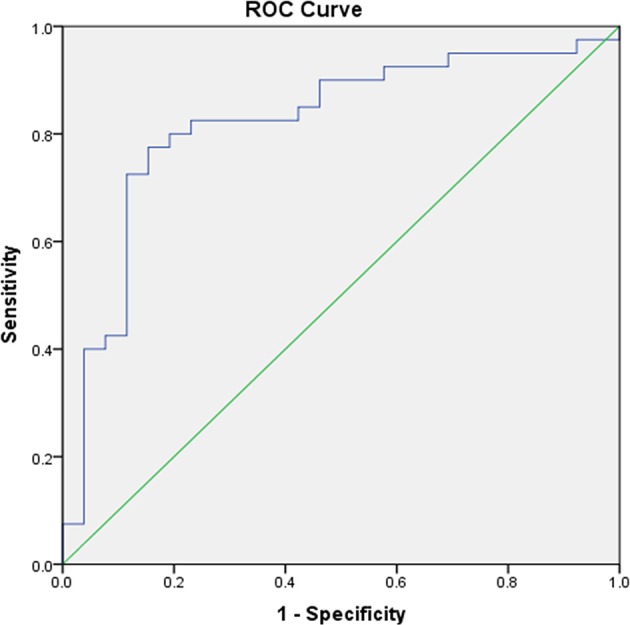
ROC analysis of the prognostic value of the combination of MR-proANP, CASP8 and NT3 on identifying patients with high recurrence risk. AUC 0.819; CI 95% (0.710–0.928) *p* < 0.001. AUC, area under the receiver operating characteristic curve; CASP8, caspase 8; CI, confidence interval; MR-proANP, mid-regional fragment of the N-terminal precursor of atrial natriuretic peptide; NT3. neurotrophin-3.

In a ROC curve analysis using presence of any arrhythmia recurrence 12 months after RFA of AF as a state variable, and MR-proANP concentration as a test variable, the produced area under the curve (AUC) was 0.693 (CI 95% 0.569–0.812; *p* = 0.005). By adding CASP8_CS_ concentration to MR-proANP concentration the AUC rose to 0.754 (CI 95% 0.640–0.868; *p* <0.001). When combining MR-proANP, CASP8 and NT3_CS_ the produced AUC was 0.819 (CI 95% 0.71–0.928; *p* < 0.001) (Figure 2).

**Figure 3:**
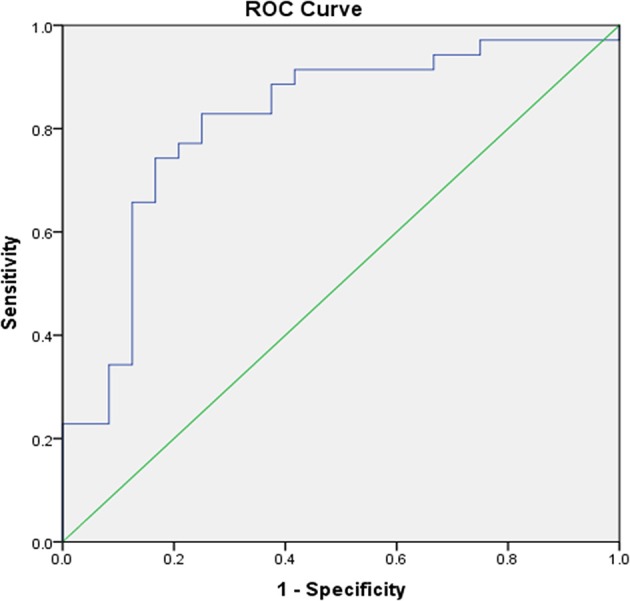
ROC analysis of the prognostic value of the combination of LA EF, CASP8, and NT3 on identifying patients with high recurrence risk. AUC 0.814; CI 95% (0.698–0.931) *p* < 0.001. AUC, area under the receiver operating characteristic curve; CASP8, caspase 8; CI, confidence interval; LA EF, left atrial emptying fraction; NT3, neurotrophin-3.

Furthermore, in another ROC curve analysis using the same state variable, and LAEF as test variable, the produced AUC was 0.685 (CI 95% 0.556–0.813; *p* = 0.011). By adding CASP8_CS_ and NT3_CS_ in the multi-predictor model the AUC rose to 0.814 (CI 95% 0.698–0.913; *p* < 0.001) (Figure 3).

## Discussion

Given the disparity in outcome after RFA for AF, the identification of predictors for a successful procedure is highly desirable. With this, it would be possible to identify the most appropriate patient categories for the procedure. In this study, we tried to identify new predictors of recurrence after a single RFA with two neurohormonal peptides, MR-proANP and NT-proBNP; two echocardiographic measures of the LA function, LAV_max_ and LA EF; and three markers of inflammation, fibrosis and apoptosis: hsCRP, NT3 and CASP8. After correcting our analyses for previously reported risk factor (Balk et al., 2010; Sultan et al., 2017) we found that patients with high concentrations of MR-proANP, CASP8, NT3, and low LA EF are at higher risk for recurrence compared to those with low concentrations of these biomarkers and high LA EF.

### Natriuretic Peptides for Predicting Recurrence After RFA

When analyzing the natriuretic peptides, we observed that MR-proANP was a statistically significant predictor for AF recurrence after RFA of AF, while NT-proBNP failed to show any significant predictive value. The pro-atrial natriuretic peptide (proANP) is stored in the atrial myocytes and splits into ANP and NT-proANP fragments on secretion secondary to increased wall tension (Mukoyama et al., 1991). Elevated ANP concentrations are frequently observed in patients with AF (Nakanishi et al., 2016) and serum ANP concentrations decrease 3 months after RFA of AF (Sacher et al., 2008). Nakanishi et al. (2016) showed that ANP concentrations before RFA were associated with LA remodeling, independent of AF type and LA volume index. Hence, ANP seems to be a good marker of AF disease progression and thus a favorable predictor of AF recurrence after RFA.

The B-Natriuretic peptide and NT-proBNP are secreted directly after synthesis from atrial and ventricular myocytes in response to increased wall tension, volume, and pressure overload (Suzuki et al., 1992; Goetze et al., 2006). In contrast to our study, a recent metanalysis by Zhuang et al. (2014) found that increased NT-proBNP concentration is associated with greater risk for recurrence after catheter ablation of AF. Interestingly, when observing the rhythm at the time for blood sampling in the included studies, it was noted to be SR for the vast majority of cases. In a previous study from our group (Zhang et al., 2016), we found that NT-proBNP concentration varies depending on actual rhythm, showing that patients in AF have considerably higher concentrations of NT-proBNP compared to those in SR, regardless of the type of the AF. In our study 27% of patients were in AF, a possible explanation for the differing results in the studies.

### LAEF for Predicting Recurrence After RFA

In our study LAEF was found to be an independent predictor of AF recurrence after RFA. On the other hand, LAV corrected for BSA failed to show any predictive value. Several studies have shown that LA function is associated with RFA success in patients with AF (Dodson et al., 2014; Chou et al., 2018) A systematic review, on the other hand, showed that LA size was a statistically independent predictor of recurrence of AF post RFA in only 4 of 20 studies (Balk et al., 2010). Similarly, a recent study by Chou et al. (Chou et al., 2018) using a multivariate analysis showed that LAEF, but not LAV, was associated with AF recurrence after RFA. This study proposed that atrial dysfunction may be an earlier indicator of AF-related changes compared to atrial enlargement (Luong et al., 2016; Chou et al., 2018).

The LAEF comprises the pump function, i.e., the contractile component of the LA, as well as the passive function, i.e., the conduit component of the LA. In patients with left ventricular diastolic dysfunction, increasing filling pressures decline LA passive function and at the same time enhances LA stretch and PV dilation, leading to an increased risk of developing AF (Chou et al., 2018). As a result of the LA passive function deterioration, the LA contractile properties are more crucial in order to maintain the LAEF (Mehrzad et al., 2014). Thus, the healthy atrial myocardium can compensate for the decline of early filling and preserve the LAEF, even though the LA can be dilated. This is an explanation why patients with LA dilation and preserved LAEF had a positive outcome after RFA of AF in the study by Chou et al. (2018).

### Inflammation/Fibrosis/Apoptosis for Predicting Recurrence After RFA

An interesting finding in our study was the fact that patients with high CASP8 and NT3 levels in CS blood had a higher risk for recurrence compared with those with low levels. On the other hand, hsCRP failed as a predictor for recurrence after RFA of AF.

CASP8 is a member of the cysteine proteases, that has shown to be proinflammatory (Liu et al., 2018) and involved in apoptosis (Kruidering and Evan, 2000). Atrial wall inflammation, fibrosis and apoptosis are processes that appear to be involved in atrial structural remodeling (Luscher, 2017). Structural remodeling delays conduction velocity (CV) by disrupting intermyocyte coupling (Burstein and Nattel, 2008). Zheng et al. (2015) showed that interstitial fibrosis was increased to different extents in AF patients, whereas more apoptotic alterations were found in the atria of AF patients. They were also able to demonstrate that angiotensin II increased the level of markers of atrial apoptosis such as CASP8. Taken together, CASP8 can be an indirect marker of angiotensin-mediated remodeling in the atria.

NT3 is a member of the family of neurotrophins (NTs). NTs are a family of structurally and functionally related growth factors that support the growth and differentiation of developing neurons. NTs have been found to regulate the fibroblast functions, including migration, cell survival, secretion of cytokines and neovascularization (Cristofaro et al., 2010; Yao et al., 2017). A recent study of Yao et al. (2017) showed that NT3 expression is enhanced in stenotic aortic valves and upregulates proliferation and collagen production in interstitial cells in the human aortic valve. Thus, NT3 could constitute a marker of fibrosis, but NT3's role in the atria remains to be studied.

Although CAPS8 and NT3 in CS blood were significant predictors of recurrence of AF after RFA, these proteins in peripheral blood did not show the same predictive value. We speculate that these differences could reflect that these proteins are involved in pathological processes primarily located in the heart in the current patients.

The mechanisms for the inflammatory process in the setting of atrial arrhythmias are important but complex and warrants further research. Many studies have examined the predictive value of hsCRP and the risk of recurrence after catheter ablation of AF (Letsas et al., 2009; Fan et al., 2012). The results of these studies are divergent. The reason for this can be the differences in study populations, and the type of AF. The results in our study are in line with the results of Fan et al. showing that hsCRP did not have any predictive value in the context of success after RFA of AF in a multivariate model (Fan et al., 2012).

### Clinical and Investigational Importance

In our study we tested different predictors as surrogate markers for the patients neurohormonal status (MR-proANP), LA function (LAEF), apoptosis (CAPS8), and fibrosis (NT3). The combination of those factors can predict recurrence after RFA of AF with high accuracy. These findings are consistent with the hypothesis that patients with better neurohormonal and mechanical LA function have a better outcome after a single RFA procedure. It also points out the need for a more thorough evaluation of AF patients in order to find the right candidates for RFA. Furthermore, CASP8 and NT3 have not been evaluated previously in this context, and further research is needed in order to understand their role in AF and as predictors of success after catheter ablation of AF.

## Limitations

This is a single-center observational cohort study with a moderate sample size. Furthermore, our sample consisted of a heterogenic group of patients including those with both paroxysmal and persistent AF, normal or reduced EF and presenting in both SR and AF. This choice was made in order to include all the categories of patients eligible for RFA in our cohort. NT3 and CASP8 were selected from a multiple biomarker panel and analyzed in a smaller group of patients in whom plasma specimen from the peripheral blood and CS was available, while hsCRP was only available in peripheral blood plasma samples. Nevertheless, no differences in the baseline characteristics was observed between this group of patients and the group of patients not included in this particular analysis (Supplementary Table 1).

Our results were based on patients undergoing RFA of AF. No other energy sources, such as cryoenergy, were used. Thus, these results can only be generalized to patients undergoing ablation using radiofrequency as energy source.

## Conclusions

Among patients who undergo their first RFA of AF, a low LAEF and elevated MR-proANP, CASP8 and NT3 were associated with an increased risk for recurrence of AF. The combination of those predictors produces a multivariate model with a high predictive value for the prediction of recurrence after RFA of AF. Patients with better LA mechanical function and lower levels of atrial neurohormones as well as of proteins related to inflammation, fibrosis and apoptosis, have a better outcome after a single RFA procedure.

## Data Availability Statement

The datasets generated for this study are available on appropriate request to the corresponding author.

## Ethics Statement

The studies involving human participants were reviewed and approved by The Regional Ethical Review Board of the Faculty of Health Sciences, Linköping, Sweden (2011/40-31, 2012/226-32). The patients/participants provided their written informed consent to participate in this study.

## Author Contributions

EC, LK, and C-JC designed the study. EC analyzed the data and drafted the manuscript. EC, LK, C-JC, UW, and J-MP interpreted the results and edited the manuscript critically. All the co-authors have read and accepted this version of the manuscript.

### Conflict of Interest

The authors declare that the research was conducted in the absence of any commercial or financial relationships that could be construed as a potential conflict of interest.
