# Supplementary material for: Echocardiographic and Biochemical Factors Predicting Arrhythmia Recurrence After Catheter Ablation of Atrial Fibrillation—An Observational Study
Source: Front Physiol. 2019 Oct 2;10:1215. doi: 10.3389/fphys.2019.01215 (PMC6783634; doi:10.3389/fphys.2019.01215)
Supplement: Supplementary file 1 [file Table_1.DOCX]

Table 1 Comparison of the baseline characteristics in patients available for the analysis of NT3 and CASP8 and those who were unavailable

| Variables | Patients included into the analysis n=75 | Patients excluded from the analysis n=114 | P value |
| --- | --- | --- | --- |
| Age mean | 59.9.5±10.1 | 61.4±10.6 | 0.338 |
| Female Gender | 24 (32 %) | 31(27.2 %) | 0.447 |
| BMI | 26.7 (20.4, 33) | 27.7 (22.2, 33.2) | 0.180 |
| Paroxysmal AF | 30 (40 %) | 41 (36 %) | 0.575 |
| Hypertension | 31 (41.3 %) | 49 (43 %) | 0.822 |
| Diabetes mellitus | 4 (5.3 %) | 12 (10.5 %) | 0.281 |
| IHD | 6 (8 %) | 10 (8.7 %) | 0.852 |
| Stroke/TIA | 4 (5.3 %) | 7 (6.1 %) | 0.817 |
| CHA2DS2VASc | 2 (0, 4) | 1.5 (0, 4.5) | 0.696 |
| CKD (GFR <60mL/min/1.73 m^2^) | 16 (21.3 %) | 24 (16.7 %) | 0.963 |
| Beta blocker | 57 (76 %) | 82 (71.9 %) | 0.535 |
| AAD | 36 (48 %) | 62 (54.4 %) | 0.330 |
| RAAS inhibitors | 66 (57.8 %) | 44 (58.6%) | 0.916 |
| Statins | 19 (25.3) | 35 (30.7) | 0.424 |
| LVEF<50% | 24 (32%) | 25 (21.9 %) | 0.122 |

**Note 1:** Normally distributed continuous data are presented as means with standard deviation and differences examined with t-test non-parametric data are presented as median values with 25^th^ and 75^th^ percentiles within brackets and tested with Mann-Whitney U test, categorical data are presented as counts with percent values within brackets and tested with chi-square test.

**Abbreviations:** AAD: antiarrhythmic drugs; AF: atrial fibrillation; BMI: body mass index; CASP8: caspase 8; CHA_2_DS_2_ VASc: congestive heart failure, hypertension, age ≥ 75, diabetes, stroke, vascular disease, gender; CKD: chronic kidney failure; LVEF: left ventricular ejection fraction; GFR: glomerular filtration rate; IHD: ischemic heart disease; NT-3: Neurotrophin 3 RAAS: renin-angiotensin II-aldosterone system
